# The Multifaceted Regulation of Mitochondrial Dynamics During Mitosis

**DOI:** 10.3389/fcell.2021.767221

**Published:** 2021-11-03

**Authors:** Evanthia Pangou, Izabela Sumara

**Affiliations:** ^1^Institut de Génétique et de Biologie Moléculaire et Cellulaire (IGBMC), Illkirch, France; ^2^Centre National de la Recherche Scientifique UMR 7104, Strasbourg, France; ^3^Institut National de la Santé et de la Recherche Médicale U964, Strasbourg, France; ^4^Université de Strasbourg, Strasbourg, France

**Keywords:** mitochondria, fission, fusion, transport, mitosis, disease

## Abstract

Mitosis ensures genome integrity by mediating precise segregation of the duplicated genetic material. Segregation of subcellular organelles during mitosis also needs to be tightly coordinated in order to warrant their proper inheritance and cellular homeostasis. The inheritance of mitochondria, a powerhouse of the cell, is tightly regulated in order to meet the high energy demand to fuel the mitotic machinery. Mitochondria are highly dynamic organelles, which undergo events of fission, fusion and transport during different cell cycle stages. Importantly, during mitosis several kinases phosphorylate the key mitochondrial factors and drive fragmentation of mitochondria to allow for their efficient distribution and inheritance to two daughter cells. Recent evidence suggests that mitochondrial fission can also actively contribute to the regulation of mitotic progression. This review aims at summarizing established and emerging concepts about the complex regulatory networks which couple crucial mitotic factors and events to mitochondrial dynamics and which could be implicated in human disease.

## Introduction

**FIGURE 1:**
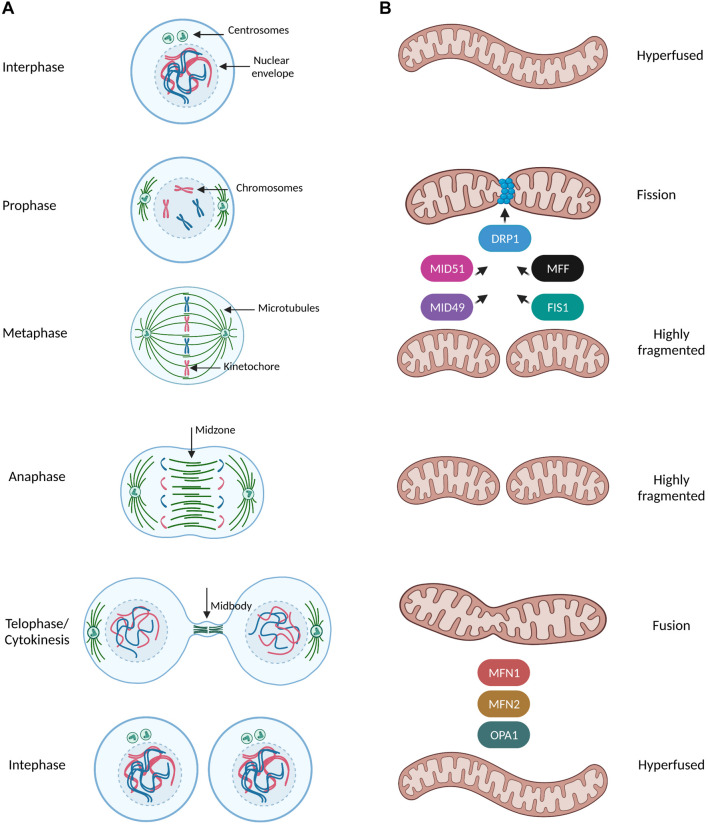
Mitochondrial dynamics during mitotic progression. **(A,B)** Simplified schematic representation of the dynamic remodeling of the mitochondrial network during mammalian cell cycle progression. During interphase (G1, S, G2 phases) mitochondria form extensively fused and interconnected networks. Prophase is the first mitotic stage and is characterized by the nuclear envelope breakdown, condensation of chromosomes and the first steps of formation of the mitotic spindle such as centrosome separation and nucleation of microtubules in MTOC. At this stage, mitochondria start to prepare for their division by recruiting the pro-fission protein DRP1 at the outer mitochondrial membrane (OMM) though interactions with its receptors (MFF, FIS1, MID49, and MID51). DRP1 oligomers constrict mitochondria and induce a highly fragmented morphology which is even more pronounced when cells reach the metaphase stage. During metaphase the spindle microtubules from the opposite spindle poles capture and bind the kinetochore region of the duplicated chromosomes and anaphase begins only when all kinetochores were attached to microtubules and all chromosomes have been properly aligned at the metaphase plate. As the sister chromatids separate from each other and are pulled toward opposite ends of the cell during anaphase, mitochondria are still fully fragmented. During telophase/cytokinesis, chromosomes start to decondense, nuclear envelope reforms, the mitotic spindle disassembles and actin forms a contractile ring in the midbody structure where final division of the cytoplasm (abscission) occurs. Mitochondria begin to form elongated structures through the coordinated actions of the pro-fusion proteins MNF1/2 at the OMM and OPA1 at inner mitochondrial membrane (INM). By the time of cytokinesis completion, mitochondria can be observed as a hyperfused interconnected network distributed into two identical daughter cells.

Mitosis is a fundamental process in eukaryotes which ensures genome integrity by elegantly coordinating the segregation of duplicated chromosomes in order to give rise to genetically identical daughter cells (McIntosh, 2016). Mitosis comprises of five stages known as prophase, prometaphase, metaphase, anaphase and telophase, where processes such as nuclear envelope (NE) breakdown, chromosome condensation, formation of the mitotic spindle, alignment of chromosomes, sister chromatids separation and finally their equal distribution to the daughter cells and reformation of two new nuclei, occur with an exquisite precision (Figure 1A). The fidelity of mitotic progression is monitored by a surveillance mechanism named the spindle assembly checkpoint (SAC) (or the mitotic checkpoint) which creates an “anaphase wait” signal and delays chromosome segregation in the presence of unstable or defective contact sites of chromosomes with the mitotic spindle, the so called microtubule-kinetochore (MT-KT) attachments (Lara-Gonzalez et al., 2021). Accumulation of segregation errors and SAC adaptation are considered as hallmarks of aneuploid cancer cells (Cohen-Sharir et al., 2021), therefore studying mitotic signaling pathways is highly important in cancer research.

*Bona fide* remodeling and distribution of subcellular organelles during cell division is equally critical for maintaining the fidelity of the genome and cellular homeostasis as recently discussed by Mascanzoni et al. (2019). Mitochondria are vital organelles, often referred to as the powerhouse of the cell in all eukaryotic organisms. Compared to other organelles, they display unique features such as a double membrane, own genetic material, the ability to produce ATP via oxidative phosphorylation (OXPHOS) and the presence of multiple mitochondrial quality control checkpoints as a means to preserve their fitness in response to distinct insult signals (Formosa and Ryan, 2018; Ng et al., 2021).

However, energy production is not the sole function of mitochondria, since they have evolved as critical regulators of various cellular processes including metabolism, apoptosis, calcium buffering and cell division. Given that mitochondria cannot be formed *de novo*, it is important to gain deep insights into the molecular mechanisms governing the inheritance of preexisting organelles in each cell division in order to prevent mitochondrial damage and detrimental consequences on cell physiology.

Mitochondria constantly undergo dynamic remodeling of their network through fusion and fission events along with cytoskeleton-based transport, revealing a complexicity behind their distinct shapes in response to different stimuli (Mishra and Chan, 2014; Figure 1A). Mitochondria fission leads to small and round mitochondria and largely relies on the recruitment of the GTPase Dynamin related protein 1 (DRP1) at the outer mitochondrial membrane by the following known receptors: mitochondrial fission factor (MFF) (Gandre-Babbe and van der Bliek, 2008), Fis1, MiD49 and MiD51 (Losón et al., 2013; Figure 1B). DRP1 assembles in high-order oligomers which progressively maturate into ring-like structures wrapping around and constricting mitochondria through selective recruitment to its different receptors at future mitochondria division sites (Fröhlich et al., 2013). On the other hand, mitochondrial fusion results in tubular and branched mitochondria and is mediated by the mitofusins MFN1 and MFN2 and by the GTPase optic atrophy 1 (OPA1) of the outer and inner mitochondrial membranes, respectively (Song et al., 2009; Figure 1B). Finally, mitochondrial transport along microtubules and actin filaments is based on the orchestrated action of the force-generating motor proteins: myosin, kinesin, and dynein (Kruppa and Buss, 2021).

Importantly, mutations which give rise to pathogenic variants of genes that regulate mitochondrial dynamics (*MFN2, OPA1, MIEF1, DNM1L, and MFF*) are causally linked to severe neurodegenerative diseases extensively discussed in the literature (Suárez-Rivero et al., 2016; El-Hattab et al., 2018; Charif et al., 2021), further highlighting the importance of maintaining mitochondrial integrity. Mutations in genes of the mitochondrial transport machinery have been described for *RHOT1* (the gene encoding Miro1 protein) and they are linked to decreased endoplasmic reticulum-mitochondrial contact sites and impaired calcium homeostasis in fibroblasts from Parkinson’s disease (PD) patients (Berenguer-Escuder et al., 2019; Grossmann et al., 2019).

Despite research efforts to elucidate the subcellular mechanisms orchestrating the complex regulatory networks linking the mitotic machinery and mitochondrial division, we still lack sufficient knowledge. Fundamental questions such as how deficiencies in the proteins that regulate mitochondrial dynamics interfere with cell physiology and hence contribute to disease development remain largely unresolved. The current review focuses on discussing established and emerging concepts about the crosstalk between the components of the mitochondrial network and the regulatory machinery of cell division.

## How Does the Mitotic Machinery Regulate Mitochondrial Homeostasis?

### Phosphorylation

**FIGURE 2:**
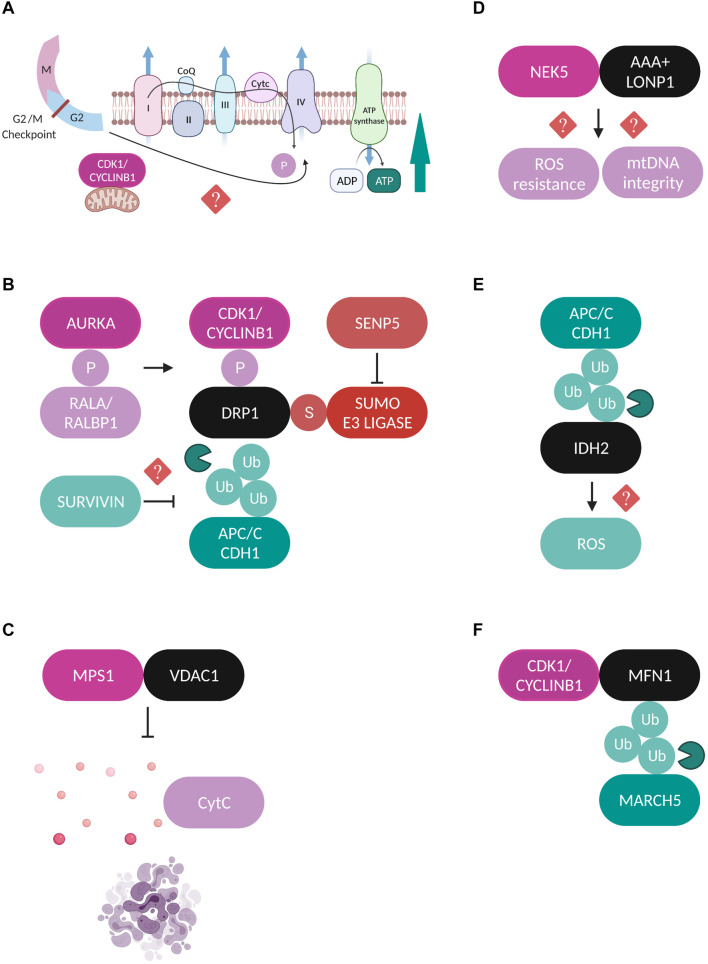
Regulation of mitochondrial proteins by mitotic factors. **(A)** A fraction of CDK1-Cyclin B1 localizes at mitochondria and is proposed to coordinate mitochondrial bioenergetics during G2/M transition. CDK1-dependent phosphorylation of substrates of the CI respiratory complex is required for CI activation and increased ATP generation through yet unidentified mechanisms (Wang et al., 2014). **(B)** Schematic representation of the different post-translational modifications which regulate DRP1 during mitosis. AURKA phosphorylates (P) RALA to enhance its interaction with its effector protein RALBP1 (Kashatus et al., 2011). This phosphorylation sequentially stimulates the phosphorylation of DRP1 by the CDK1-Cyclin B1 complex which promotes its mitochondrial recruitment (Taguchi et al., 2007; Kashatus et al., 2011). Moreover, APC/C^*CDH*1^ regulates the stability of DRP1 through direct ubiquitylation (Ub) and proteasomal degradation (Horn et al., 2011). Survivin has been suggested to facilitate DRP1 recruitment to mitochondria possibly through repressed APC/C^*CDH*1^ expression, but the exact mechanism is unclear (Hagenbuchner et al., 2013). Finally, SENP5 desumoylates DRP1 and promotes its oligomerization (Zunino et al., 2009). All the above described modifications lead to enhanced DRP1-mediated mitochondrial fission and accurate chromosome segregation. **(C)** MPS1 is recruited to mitochondria through binding to VDAC1 and inhibits the VDAC1-dependent cytochrome c release and subsequent apoptosis (Zhang et al., 2016). **(D)** NEK5 binds to the AAA+ mitochondrial protease LONP1 and this interaction is correlated with increased mtDNA integrity and ROS resistance, but the exact mechanisms are not yet defined (Ferezin et al., 2021). **(E)** APC/C^*CDH*1^ ubiquitylates the NADPH-producing enzyme IDH2, leading to elevated ROS production, although no direct mechanism has been described (Lambhate et al., 2021). **(F)** CDK1/Cyclin B1 complex binds to MFN1 and this interaction triggers its ubiquitylation by the mitochondrial E3 ubiquitin ligase MARCH5 and proteasomal degradation (Park and Cho, 2012).

Protein kinases are the most well studied category of mitotic factors that have been identified to play crucial roles in regulating mitochondrial dynamics, inheritance and function in mammalian cells. Among them, a lot of research has focused on the cyclin dependent kinase 1 CDK1-Cyclin B1 complex or maturation promoting factor (MPF), a major cell division kinase which controls the transition from G2 phase into mitosis (Łukasik et al., 2021). Interestingly, an enzymatically active subfraction of CDK1-Cyclin B1 can be localized at mitochondria during G2/M phase, acting as a coordinator of mitochondrial bioenergetics to meet the high energy demand required to fuel this cell cycle transition (Wang et al., 2014). Phosphoproteomics analysis revealed a cluster of 52 mitochondrial proteins as potential CDK1-Cyclin B1 phosphorylation targets, including key proteins of the OXPHOS machinery and in particular substrates of the respiratory complex I (CI). CDK1-dependent phosphorylation of these proteins is indispensable for CI activation and enhances ATP generation, linking mitotic progression to mitochondrial activity (Figure 2A). Although the exact mechanism which regulates the mitochondrial influx of CDK1-Cyclin B1 remains unknown, the authors suggest that it could potentially be under the control of a mitochondrial target sequence (MTS) identified at the N-terminus of Cyclin B1, or via its interaction with some chaperone proteins.

Moreover, yeast studies revealed that CDK1 directly phosphorylates the translocate precursor tom6 specifically during G2/M transition, stimulating the assembly of the protein import channel tom40 (Harbauer et al., 2014). This phosphorylation induces the mitochondrial import of the fusion proteins fzo1 and mgm1 (corresponding to the human homologs MFN2 and OPA1, respectively) leading to elevated mitochondrial respiratory activity during mitosis.

Furthermore, CDK1-Cyclin B1 phosphorylates DRP1 on Ser 616 to stimulate its mitochondrial fission activity specifically in mitosis (Taguchi et al., 2007). This modification is enhanced through upstream phosphorylation of the small Ras-like GTPase RALA on Ser 194 by Aurora A kinase (AURKA) which drives RALA localization and its subsequent interaction with its effector protein RALA-binding protein 1 (RALBP1) at the mitochondrial surface (Kashatus et al., 2011). The RALA/RALBP1 active complex facilitates the phosphorylation of DRP1 on Ser 616 by the CDK1-Cyclin B1, thereby promoting DRP1 recruitment to mitochondria, fission, and proper segregation of mitochondria during mitosis (Figure 2B). Despite being originally considered as a mitotic kinase and in addition to its role in promoting mitochondrial fission in mitotic cells, AURKA’s role in regulating mitochondrial dynamics is also extended to interphase in a RALA-independent mechanism. An active pool of AURKA is localized at mitochondria through an atypical N-terminus mitochondrial targeted sequence (MTS) both in drosophila and in human cells throughout the cell cycle (Bertolin et al., 2018; Grant et al., 2018). Interestingly, the effect of AURKA on mitochondrial morphology in interphase depends on its expression levels. Under physiological conditions AURKA promotes fission while overexpression of AURKA leads to hyperfusion due to direct inhibitory interactions with the pro-fission proteins DRP1 and MFF (Bertolin et al., 2018).

Another example of communication between mitochondria and the mitotic phosphorylation machinery is the recruitment of the mitotic kinase monopolar spindle (MPS1) to the mitochondrial compartment through its binding to the voltage dependent anion channel protein (VDAC1) (Zhang et al., 2016). MPS1 is a key component of the SAC which represents the major mitotic surveillance mechanism (Pachis and Kops, 2018). The interaction of MPS1 with VDAC1 is strongest during mitosis and contributes to increased cell viability by inhibiting the VDAC1-dependent cytochrome c release and subsequent apoptosis (Figure 2C; Zhang et al., 2016). Recently, the Nima-related kinase 5 (NEK5) was added to the list of mitotic kinases that can localize and exert new functions at mitochondria. NEK5 was found to interact and co-localize with the AAA+ mitochondrial Lon protease (LONP1) in proximity ligations assays (PLA) and the authors suggest that a fraction of NEK5 can be localized at the mitochondrial nucleoids through a signaling axis that involves the transcription factor A mitochondrial (TFAM) (Ferezin et al., 2021). The binding between NEK5 and LONP1 was correlated with increased mitochondrial DNA (mtDNA) integrity, resistance to oxidative damage and positive regulation of mtDNA repair genes, however, the exact molecular mechanisms are not yet defined (Figure 2D).

### Ubiquitylation and Other Mitotic Signaling

The anaphase-promoting complex/cyclosome (APC/C) is the E3 ubiquitin ligase and the central mediator of the ubiquitin-dependent degradation of dozens of substrates during mitotic exit through the coordinated actions of its co-activators CDC20 and CDH1 (Yamano, 2019). As such, it has also been reported to play roles in the regulation of mitochondrial morphology by contributing to the maintenance of a dynamic balance between fission and fusion during mitotic exit. For example, the stability of the major mitochondrial pro-fission protein DRP1 is under the APC/C^*CDH*1^ control, which mediates the ubiquitylation and subsequent proteasomal degradation of DRP1 by binding to its destruction box (D-box) motif (Figure 2B; Horn et al., 2011). Moreover, the extensive mitochondrial fragmentation that is caused by CDH1 deficiency during mitotic exit, can be entirely rescued upon AURKA inactivation, suggesting that degradation of AURKA by APC/C^*CDH*1^ is a crucial step for the post-mitotic reassembly of the mitochondrial network (Abdelbaki et al., 2020). Finally, a recent study proposed a new role for APC/C^*CDH*1^ in the regulation of mitochondrial function during mitosis where it was shown that APC/C^*CDH*1^ ubiquitylates isocitrate dehydrogenase 2 (IDH2), a major NADPH-producing enzyme in mitochondria, thus indirectly enhancing the production of reactive oxygen species (ROS) during mitosis, although the exact mechanism is not yet clear (Figure 2E; Lambhate et al., 2021).

Sentrin specific protease 5 (SENP5) is a sumo protease with essential roles in mitosis and its depletion leads to cytokinesis failure (Di Bacco et al., 2006). Translocation of SENP5 from the nucleoli to the mitochondria at G2/M transition interferes with the sumoylation state of DRP1, promoting its oligomerization and subsequent mitochondrial fission during mitosis (Figure 2B; Zunino et al., 2009). Survivin is a protein known as part of the chromosomal passenger complex (CPC), mediating the targeting of CPC to the centromeres and contributing to the proper alignment of mitotic chromosomes. While accumulating evidence suggests that mitochondria-localized survivin is a unique cancer feature, the way it affects the metabolic reprogramming of cancerous cells and whether this is a cell cycle-dependent event is still controversial (Wheatley and Altieri, 2019). Survivin is also implicated in mitochondrial dynamics by facilitating the recruitment of DRP1 to mitochondria and promoting fission, a process correlated with repressed APC/C^*CDH*1^ expression, although the exact mechanism is not yet known (Figure 2B; Hagenbuchner et al., 2013). Finally, to our knowledge there are currently no studies addressing how fusion-related proteins are regulated in a cell cycle-dependent manner, with only one study showing that MFN1 is targeted for proteasomal-dependent degradation specifically during G2/M transition by the mitochondrial E3 ubiquitin ligase MARCH5 (Park and Cho, 2012). The authors propose that MFN1 degradation is the result of its interaction with CDK1/Cyclin B1 complex, but the effects of this modification on chromosome segregation were not further investigated (Figure 2F). Regarding the cell cycle regulation of OPA1 one speculation might be that since OPA1 requires MFN1 in order to perform its fusion-promoting activity (Cipolat et al., 2004), then OPA1-mediated fusion could be indirectly inactivated during mitosis due to the reported MFN1 degradation. It would be important to fill in this research gap in the future and to try to understand the molecular mechanisms which contribute to the regulation of mitochondrial fusion machinery during mitotic progression.

## How Do Mitochondrial Function and Mitochondrial Inheritance Regulate Mitosis?

### Mitochondrial Dynamics

**FIGURE 3:**
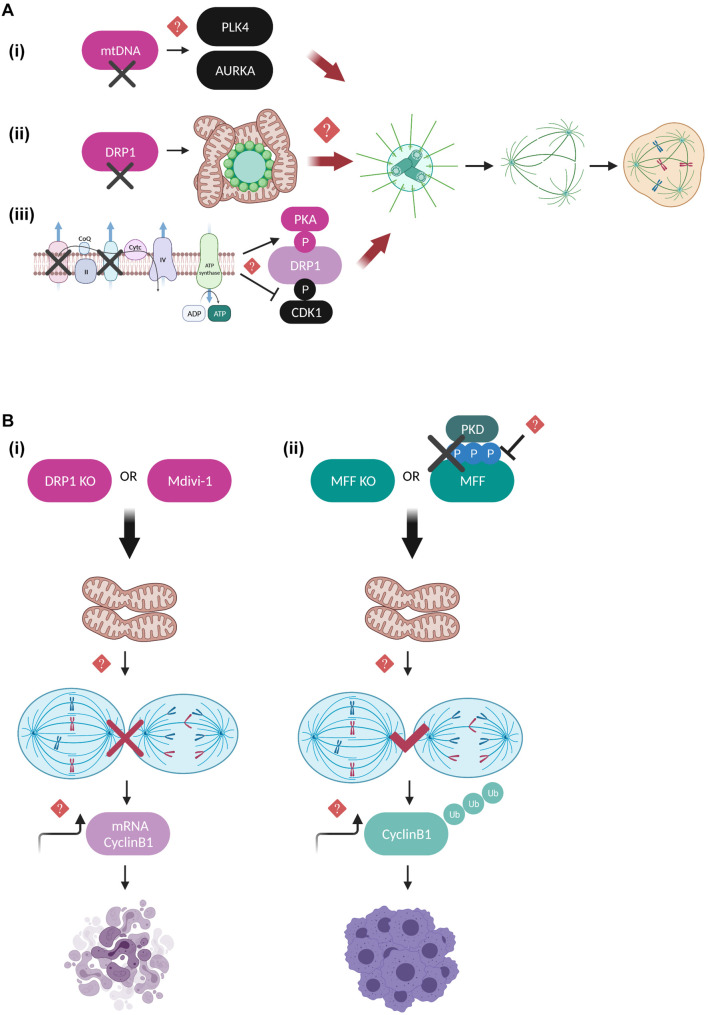
Regulatory mechanisms coupling mitotic progression to mitochondrial dynamics. **(A)** Schematic representation of the mitochondrial pathways that are suggested to lead to aberrant number of centrosomes, perturbed mitotic spindle architecture and segregation errors when deregulated. mtDNA-depleted cells display elevated expression levels of PLK4 and AURKA kinases, possibly leading to their continuous activation and uncontrolled phosphorylation of their mitotic spindle substrates (Donthamsetty et al., 2014). Moreover, DRP1 depletion leads to hyperfused mitochondrial networks that interfere with the MTOC and result in defective microtubule nucleation, although it is not clear whether this is DRP1-dependent or a general characteristic of impaired mitochondrial fission (Qian et al., 2012). Finally, inhibition of the mitochondrial respiratory complexes I and III leads to enhanced PKA-mediated and reduced CDK1-mediated DRP1 phosphorylation, further promoting mitochondrial hyperfusion and multipolar spindle formation (Ko et al., 2021). However, the exact molecular mechanisms linking mitochondrial function to the maintenance of centrosome and mitotic spindle homeostasis remain unknown. **(B)** Defects in the mitochondrial fission machinery are coupled to mitotic progression and interestingly have entirely different outcomes toward cell division and cell fate depending on whether mitochondrial hyperfusion occurs downstream of DRP1 depletion/inhibition or downstream of MFF depletion and/or inhibition of PKD-mediated MFF phosphorylation. DRP1 downregulation leads to prolonged mitotic arrest and apoptotic cell death, thereby protecting cells from dividing in the presence of dysfunctional mitochondria (Díaz-Martínez et al., 2014; Peña-Blanco et al., 2020). On the other hand, MFF downregulation of MFF lacking PKD-mediated phosphorylation leads to anaphase initiation in the presence of chromosome segregation errors, mitotic slippage and polyploidy (Pangou et al., 2021). In both cases, imbalance of Cyclin B1 translation and degradation rates is suggested to be mechanistically involved, however, the direct implication of Cyclin B1 in the above described pathways requires further investigation.

The interactive regulation of mitochondria by the mitotic machinery is undoubtedly reciprocal. Compelling evidence suggests that functional mitochondria are required to ensure mitotic fidelity thanks to their role in maintaining centrosome homeostasis (detailed illustration in Figure 3A). Cells depleted of mtDNA are characterized by severe defects in centrosome duplication and spindle architecture, as well as elevated levels of key centrosome integrity regulators such as Polo like kinase 4 (PLK4) and AURKA, however, a detailed mechanism has not yet been described (Figure 3A; Donthamsetty et al., 2014). Extensive research has also focused on elucidating the direct function of mitochondrial fission and fusion factors on mitotic progression, with DRP1-related studies being in the spotlight. Hyperfusion caused by DRP1 deficiency induces aberrant distribution of the mitochondrial network around the microtubule organizing center (MTOC), which triggers centrosome overduplication, mitotic spindle defects, chromosomal instability, replication stress and G2/M arrest (Figure 3A; Qian et al., 2012). Abnormal centrosome amplification can often induce mitotic catastrophe, a type of mitotic cell death in cells lacking functional apoptotic pathways, usually after delayed DNA damage induced by ionizing irradiation (Sia et al., 2020). In this context, DRP1 depletion is suggested to rescue both the aberrant centrosome numbers and the mitotic catastrophe-associated phenotypes following irradiation, possibly due to perturbed APC/C-mediated Cyclin B1 destruction that leads to persistent Cyclin B1 activation in irradiated DRP1-depleted cells (Yamamori et al., 2015). Additional studies from the same group proposed that mitochondrial fission mediated by DRP1 and FIS1 enhances intracellular Ca^2+^ levels after x-irradiation and is partially responsible for the induction of mitotic catastrophe in mouse breast cancer cells (Bo et al., 2020). A new study provided some additional evidence on the role of DRP1 in centrosome function and bipolar spindle assembly that ensure genome integrity (Ko et al., 2021). The authors claim that under conditions of blocking the mitochondrial electron transport chain (ETC) complexes I and III or DRP1 activity during mitosis, mitophagy and formation of multipolar spindles are enhanced due to a switch in DRP1 phosphorylation state, with Protein kinase A (PKA) phosphorylation being the predominant compared to the one mediated by CDK1 (Figure 3A). The above results appear to be contradictory to the notion that during interphase DRP1 is subjected to inhibitory phosphorylation on Ser 637 by PKA (Chang and Blackstone, 2007), while during mitosis CDK1-dependent activatory phosphorylation of DRP1 on Ser 616 is the one driving mitochondrial fission (Taguchi et al., 2007; Kashatus et al., 2011). The crosstalk between the DRP1 phosphorylation sites was recently addressed in an *in vivo* mice study, not in the context of cell division but rather on how it can influence metabolic adaptation (Valera-Alberni et al., 2021). The authors suggest that in mouse tissues, DRP1 phosphorylation on Ser 637 (S600 in mice) by PKA and phosphorylation on Ser 616 (S579 in mice) by CDK1 are both required to promote mitochondrial fission and that S637 acts upstream of S616 to trigger its activation. Moreover, PKA-dependent DRP1 phosphorylation impairs the mitochondrial respiratory capacity in multiple tissues and renders mice more susceptible to glucose intolerance induced by high fat diet. Overall, the above studies provide exciting insights how metabolic cues are coupled to cell cycle pathways by fine-tuning mechanisms of mitochondrial dynamics.

Quantitative proteomics and genome-wide siRNA screening studies have further confirmed the importance of DRP1 in regulating cell fate decisions under conditions of prolonged mitotic arrest (Díaz-Martínez et al., 2014; Peña-Blanco et al., 2020). DRP1-depleted cells are protected from exiting mitosis in the presence of dysfunctional mitochondria and instead shift toward accelerated apoptotic cell death and mitophagy (Peña-Blanco et al., 2020), while chemical inhibition of DRP1 by Mitochondrial division 1 inhibitor (Mdivi-1) (Cassidy-Stone et al., 2008) inhibits SAC adaptation and aberrant cytokinesis possibly by indirectly affecting the translation rate of Cyclin B1 (Figure 3B; Díaz-Martínez et al., 2014). The above results should nevertheless be interpreted with caution since Mdivi-1 has been shown to greatly affect mitochondrial metabolic activity by acting as a mitochondrial complex I inhibitor without impairing DRP1 GTPase activity (Bordt et al., 2017), hence additional studies including genetic evidence for DRP1 would be required to fully support these conclusions.

Only a limited number of studies has addressed so far the functional interplay of DRP1 receptors with the mitotic machinery. Recently, our group provided evidence that MFF, the predominant DRP1 receptor in mammalian cells (Otera et al., 2010), is directly phosphorylated by protein kinase D (PKD) on serines 153, 175, and 275 specifically during mitosis in order to couple mitotic mitochondrial fission to fidelity of chromosome segregation (Pangou et al., 2021). These phosphorylation events collectively act as a protective mechanism from SAC adaptation in the presence of chromosome bi-orientation errors, since cells with a defective PKD-MFF pathway are characterized by an extensively fused mitochondrial network, premature anaphase initiation, mitotic exit without proper chromosome segregation (mitotic slippage), polyploidy and finally, reduced long-term proliferation capacity (Figure 3B). Importantly, our findings demonstrated that PKD phosphorylation does not interfere with the ability of MFF to induce mitochondrial fragmentation in interphase, further suggesting that phosphorylated MFF represents the main signaling trigger for mitotic mitochondrial fission and its function may not be compensated by other existing mitochondrial fission receptors. Although the implication of FIS1 in mediating mitochondrial fission by acting as an adaptor for DRP1 in mammalian cells has been recently questioned (Osellame et al., 2016; Kleele et al., 2021), previous studies had investigated its role in mitotic progression as a DRP1 receptor. Inhibition of mitochondrial fission upon FIS1 downregulation blocked mitotic entry and prolonged G2/M arrest through a pathway that was not dependent on CDK1 activity but instead involved upstream mitotic regulators such as Polo like kinase 1 (PLK1) and the transcription factor Forkhead Box M1 (FOXM1) (Lee et al., 2014). Moreover, while mitochondrial fission has been ultimately associated with the recruitment of DRP1 to the outer mitochondrial membrane, an additional Dynamin GTPase family member has also been identified as a critical component of the mitochondrial division machinery. Dynamin-2 (DYN2), a protein mostly known for its role in vesicle trafficking and endocytosis (González-Jamett et al., 2013), is suggested to cooperate with DRP1 in a sequential mitochondrial membrane constriction model where DYN2 acts downstream of DRP1 assembly to further drive membrane constriction and completion of mitochondrial fission process (Lee et al., 2016). However, contradictory studies performed in cells lacking all Dynamin isoforms argue that DYN2 is dispensable for both mitochondrial and peroxisomal fission, while DRP1 is the determining factor controlling organelle fission (Kamerkar et al., 2018; Fonseca et al., 2019). These opposing observations could potentially be explained by the different experimental conditions used in each study, but also by the fact that DNM2 might be involved in other processes unrelated to DRP1-dependent mitochondrial fission, such as the scission of mitochondria-derived vesicles and/or microtubule-related trafficking. Nevertheless, it would be exciting to further dissect whether DYN2 might be involved in mitochondrial dynamics in a cell cycle dependent manner given that DYN2 has been reported to be subjected to inhibitory phosphorylation at the onset of mitosis by CDK1 at Ser 764, which interferes with its midbody localization leading to cytokinesis failure and increased number of multinucleated cells (Chircop et al., 2011). Apart from the general notion that DYN2 mutations give rise to severe neuromuscular disorders (González-Jamett et al., 2013), it was recently observed that Schwann cells ablated for DYN2 display cell cycle progression and cytokinesis defects associated with severe demyelination and peripheral neuropathy development (Gerber et al., 2019). Hence, it would be worth investigating whether CDK1-dependent DYN2 phosphorylation is implicated in coordinating the final steps of mitochondrial division by mediating the fine-tuning of the abscission checkpoint regulating cytokinesis fidelity (Nähse et al., 2017). Such research could possibly bring up novel signaling pathways to be therapeutically targeted in mitochondrial diseases of the peripheral nervous system.

### Other Mitochondrial Factors

Fission and fusion are not the only processes which couple mitochondria to cell division. Critical mitotic factors and pathways are under the strict regulation of additional mitochondrial proteins which often tend to localize at mitotic structures. One example that has been extensively studied is the mitotic signaling mediated by the PTEN-induced serine/threonine kinase 1 (PINK1) and the E3 ubiquitin ligase PARKIN. While, the PINK/PARKIN pathway has mostly been studied for its involvement in Parkinson disease and in mitochondrial quality control through mitophagy, several lines of evidence have proven that PINK/PARKIN activation has also prominent roles in driving mitochondrial dynamics by activating pro-fission and inactivating pro-fusion proteins, research that is described in detail in the recent review article (Ge et al., 2020).

**FIGURE 4:**
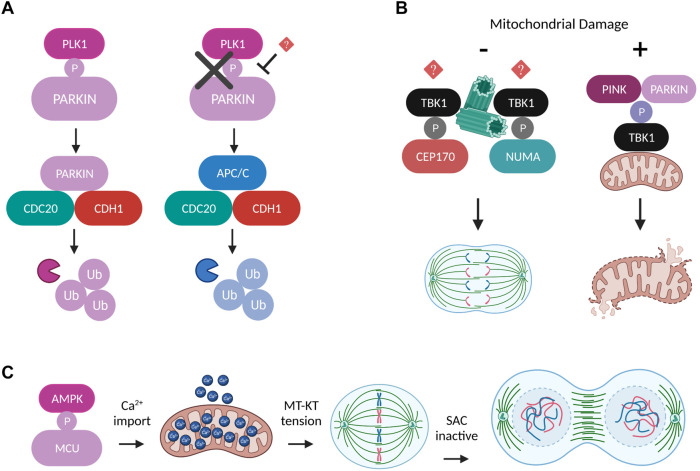
Regulation of mitosis by other mitochondrial factors. **(A)** Schematic representation of two alternative ubiquitylation pathways which can work independently and in parallel to mediate degradation of mitotic substrates. The E3 ligases PARKIN and APC/C compete for binding to the coactivators CDC20 and CDH1 in a process dictated by the ability of PLK1 to directly phosphorylate PARKIN at the onset of mitosis (Lee et al., 2015). **(B)** Under normal conditions TBK1 is recruited to the centrosomes through unknown mechanisms, where it phosphorylates NUMA and CEP170 in order to promote microtubule stability and mitotic spindle integrity (Pillai et al., 2015). However, upon conditions of mitochondrial damage, the phosphorylation of TBK1 by PINK/PARKIN induces its translocation to the damaged mitochondria and perturbs TBK1’s mitotic functions until the damaged organelles are removed (Sarraf et al., 2019). **(C)** AMPK-mediated MCU phosphorylation boosts Ca^2+^ import into mitochondria and leads to elevated ATP production that is required to generate the amount of tension needed between MT-KT to drive SAC satisfaction and mitotic progression (Zhao et al., 2019).

PARKIN localizes at the centrosomes throughout mitosis and expression of its C-terminal domain acts as a dominant negative SAC regulator, leading to mitotic slippage, multinucleated cells and chromosomal instability (Chen et al., 2012). Later on, these defective PARKIN-associated mitotic phenotypes were further confirmed and molecularly dissected in a study demonstrating that PARKIN interacts with the APC/C coactivators CDC20 and CDH1 to mediate degradation of key mitotic substrates independently of APC/C activity, serving as an alternative parallel E3 ligase mitotic pathway (Lee et al., 2015). Importantly, PARKIN is directly phosphorylated on Ser 378 by PLK1 at the onset of mitosis and disruption of this activatory phosphorylation abolishes the complex formation between PARKIN and CDC20, and consequently PARKIN-mediated mitotic ubiquitylation (Figure 4A). Furthermore, PINK1 was identified as a critical factor that couples normal cell cycle progression with the regulation of mitochondrial dynamics (O’Flanagan et al., 2015). The authors show that PINK1 kinase activity is indispensable for mitotic progression, cytokinesis completion and cell cycle exit at G0/G1 and they provide substantial evidence for a causal link with DRP1 impaired pro-fission activity. More specifically, PINK-depleted cells are multinucleated with excessively fragmented mitochondria due to persistent CDK1-dependent DRP1 phosphorylation resulting in its increased mitochondria recruitment. A recent study using mouse and fly genetics unraveled the Tank Binding Kinase 1 (TBK1) as a novel molecular link between PINK/PARKIN-mediated mitophagy and mitosis (Sarraf et al., 2019). The authors describe that upon conditions of mitochondrial damage during G2/M transition, PINK/PARKIN induces the translocation of active TBK1 from the centrosomes to the damaged organelles, thus perturbing TBK1 function in facilitating mitotic progression and ensuring that cell division is blocked until mitochondrial clearance is achieved (Figure 4B). TBK1 localizes at centrosomes where it binds to and phosphorylates the mitotic proteins Nuclear Mitotic Apparatus Protein 1 (NuMA) and Centrosomal Protein 170 (CEP170), thereby promoting microtubule stability (Pillai et al., 2015), however, the mechanisms upstream of TBK1 mitotic activation and its targeting to key mitotic structures remain elusive. Intriguingly, TBK1 is also implicated in shaping mitochondrial dynamics through a signaling pathway linked to nutrient sensing which involves direct phosphorylation of DRP1 on Ser 412 and Ser 684 preventing its oligomerization and pro-fission activity (Chen et al., 2020). Based on the aforementioned literature and considering that TBK1 activation can also occur independently of PINK/PARKIN and promote mitophagy via the AMP-activated protein kinase (AMPK)-MFF mitochondrial fission signaling axis (Toyama et al., 2016; Seabright et al., 2020), it is tempting to speculate that TBK1 might be subjected to distinct regulatory mechanisms depending on the cell cycle stage and energy availability, and dictate diverse cell fates in order to maintain the fidelity of mitochondrial division. Finally, within the context of characterizing mechanisms which mediate mitochondrial metabolic adaptations in response to energy sensing during cell cycle progression, a new study suggested that activatory phosphorylation of the mitochondrial Ca^2+^ uniporter (MCU) on Ser 57 by AMPK is vital for boosting Ca^2+^ import and therefore ATP production in mitochondria specifically during mitosis (Zhao et al., 2019). MCU depletion or loss of its AMPK-mediated phosphorylation results in severe delay of anaphase onset due to impaired MT-KT tension and SAC potentiation (Figure 4C).

## How Is Mitochondrial Transport Regulated During Mitosis?

Cytoskeleton-based transport represents another important aspect of mitochondrial dynamics, but the exact mechanisms that govern the intracellular mobility and redistribution of the mitochondrial network during mitosis in mammalian cells remain elusive (Kanfer and Kornmann, 2016). Given that cellular architecture is subjected to major remodeling during cell division, deciphering how mitochondria interconnect to spindle microtubules and actin filaments is critical for understanding the mechanisms of their inheritance. There are three classes of motor proteins which associate with mitochondria in order to regulate their transport in an ATP-dependent manner: the actin-based myosins as well as the kinesins and dynein that can regulate the microtubule-based mobility (Kruppa and Buss, 2021). However, how exactly they couple their diverse functions during mitosis to mitochondrial dynamics has not been extensively described yet. Super resolution microscopy has enabled us to monitor in detail the spatiotemporal association of mitochondria with microtubules and with actin of the contractile ring during cytokinesis, revealing for example that mitochondria connect to astral microtubules of the mitotic spindle followed by their delivery to the cleavage furrow through a Miro-KIF5B (kinesin 1)-based mechanism (Lawrence et al., 2016).

**FIGURE 5:**
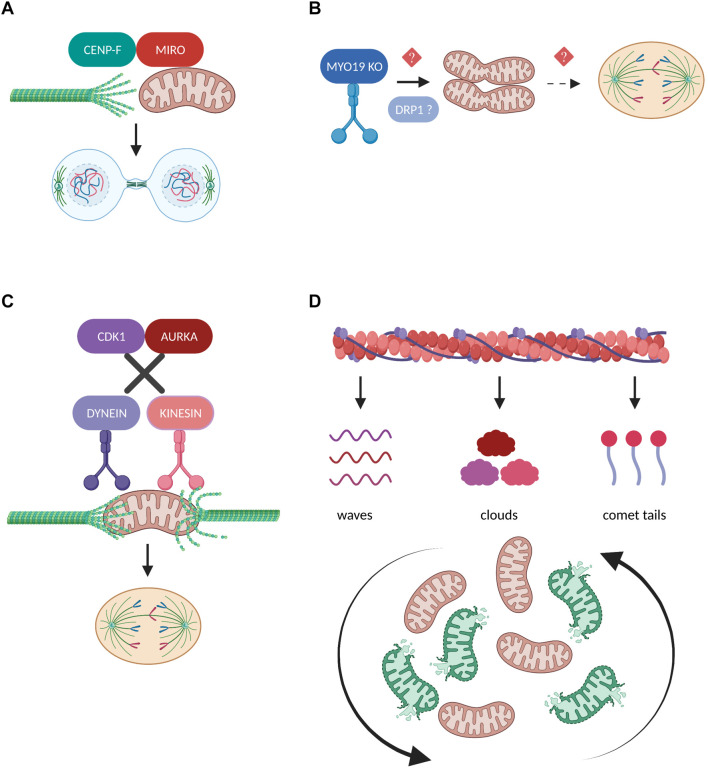
Regulation of mitochondrial transport in mitosis. **(A)** During cytokinesis, the mitochondrial adaptor protein MIRO recruits a fraction of CENP-F to mitochondria in order to promote the organelles’ association with the growing tips of microtubules. The interaction between MIRO and CENP-F is required for the efficient microtubule-based transport of mitochondria and their equal inheritance into the two daughter cells (Kanfer et al., 2015, 2017). **(B)** Depletion of the actin-based motor protein MYO19 leads to mitochondrial misplacement which blocks the assembly of the cytokinetic machinery and results in segregation errors (Rohn et al., 2014). Moreover, MYO19 is suggested to be required for the DRP1-dependent mitotic mitochondrial fragmentation (Majstrowicz et al., 2021), but whether these two pathways downstream of MYO19 could be mechanistically linked remains to be investigated. **(C)** Disruption of the phosphorylation cascades downstream of CDK1 and AURKA leads to the continuous mitochondrial recruitment of the motor proteins Dynein and Kinesin, respectively. As a result, mitochondria fail to detach from the microtubules and impair mitotic spindle dynamics and mitotic progression (Chung et al., 2016). **(D)** The inheritance of mitochondria during mitosis depends on their dynamic association with three distinct actin assemblies namely actin cables, actin clouds and actin comet tails. These interactions differentially affect the range of mitotic mitochondrial movements and are indispensable for reshuffling and redistributing healthy and damaged organelles (Moore et al., 2021).

Miro proteins are transmembrane, calcium-binding, atypical Rho GTPases with an established role as mitochondrial adaptors that link mitochondrial trafficking to microtubules through the coordinated activities of kinesin and dynein motors (Kanfer and Kornmann, 2016). Several studies from the Kornmann laboratory focused on characterizing the unexpected interaction between Miro and Centromere protein F (CENP-F), a microtubule binding factor required for proper kinetochore function and chromosome segregation, in the context of mitochondrial microtubule-based transport (Kanfer et al., 2015, 2017; Peterka and Kornmann, 2019). During cytokinesis, Miro recruits a subpopulation of CENP-F to mitochondria to promote the organelles’ association with the growing tips of microtubules (Figure 5A). Depletion of either Miro or CENP-F results in mitochondria clustering in the perinuclear region which then fail to be efficiently spread and transported toward the cell periphery in the daughter cells, thus impairing proper mitotic redistribution of the mitochondrial network (Kanfer et al., 2015). *In vitro* reconstitution of this pathway suggested that the C-terminus domain of CENP-F is responsible for both its binding to Miro as well as for its ability to transport mitochondrial cargos along microtubule tips independently of the tubulin polymerization state (Kanfer et al., 2017). Subsequently, an *in vivo* study revealed that the Miro-dependent mitochondrial function of CENP-F is cell type-specific and that CENP-F carrying the F2872A mutation fails to interact with Miro or to be recruited to mitochondria, nevertheless retaining the ability to exert its mitotic functions (Peterka and Kornmann, 2019). Intriguingly, mice bearing the CENP-F F2872 mutation alleles are viable and fertile despite displaying irregular trafficking and distribution of their mitochondria, further suggesting a tissue-specific function for the Miro/CENP-F signaling axis. Future in-depth phenotypical dissection studies will provide more insight on the unexpected role of CENP-F in the mitochondrial transport. The emerging role of Miro proteins as critical regulators of cell division through regulation of mitochondrial positioning was further confirmed by a genetic analysis showing that mice lacking Miro are characterized by impaired chromosome segregation and slow mitotic rate which partially contributes to their embryonic lethality (López-Doménech et al., 2018). Collectively, the above findings clearly indicate that functional Miro proteins are non-redundant for fine-tuning mitochondrial mitotic transport and inheritance, thereby ensuring genome integrity.

The unconventional myosin 19 (Myo19) is an actin-based motor protein whose protein stability and mitochondrial recruitment depends on its binding and interaction with the mitochondrial transmembrane proteins Miro (López-Doménech et al., 2018; Oeding et al., 2018). Myo19 depletion leads to cytokinesis failure and multinucleation likely not due to interference with the rate of mitochondrial fission and fusion, but because of misplacing mitochondria during metaphase to anaphase transition, thus generating a physical barrier against the proper assembly of the cytokinetic machinery (Figure 5B; Rohn et al., 2014). Remarkably, Myo19 is also suggested to regulate mitochondrial dynamics specifically in mitotic cells, where its deficiency leads to impaired mitochondrial fragmentation in prometaphase despite the fact that DRP1 total protein levels are elevated, further confirming the role of Myo19 in coupling mitochondrial division to mitotic progression (Majstrowicz et al., 2021). The authors suggest that no difference was observed regarding the mitotic phosphorylation status of DRP1 known to regulate its pro-fission activity, however, the presence of fused mitochondria implies that future studies should address the involvement of Myo19 or in general of actin remodeling in modulating DRP1 oligomerization, interaction with its receptors and subsequent recruitment to mitotic mitochondria.

In contrast to studies supporting that mitochondrial trafficking during cell division is mediated through microtubule- and/or actin-based active transport, some evidence also demonstrates the existence of a passive model for mitochondrial positioning and inheritance (Chung et al., 2016). The authors illustrate that during normal mitotic progression, mitochondria are detached from the microtubules and localize at the periphery of the mitotic spindle through the coordinated actions of CDK1 and AURKA which phosphorylate several substrates, thereby inducing dynein and kinesin removal from the mitochondrial surface, respectively. However, when motor proteins are forcefully recruited to mitochondria and remain attached to their adaptor complexes, mitochondria are connected to the spindle microtubules and interfere with mitotic progression which eventually leads to their asymmetric inheritance (Figure 5C). Given the important role of CDK1 and AURKA in promoting mitotic mitochondrial fission (Kashatus et al., 2011), it would be fascinating to investigate whether phosphorylation cascades could determine the choice for passive or active mitochondrial transport depending on the fine-tuned balance of mitochondrial dynamics during cell division.

A recent scientific breakthrough study challenged our view on how actin filaments interact with mitochondria to ensure their equal partitioning during symmetrical cell division, proving that this is far from a passive process but rather depends on multiple, parallel coordinated events (Moore et al., 2021). The authors describe that mitochondria can associate with three distinct actin assemblies namely actin cables, actin clouds and actin comet tails and display unique motility signatures which can vary from heavily constricted to unlimited mitochondrial movements during mitosis (Figure 5D). These dynamic actin waves are indispensable to reshuffle the positioning of mitochondria in order to boost the redistribution of locally damaged organelles, thus contributing to faithful mitochondrial inheritance. Furthermore, actin polymerization/depolymerization cycles have been reported to locally finetune the balance between mitochondrial fission and fusion in interphasic cells and DRP1 is required to efficiently rearrange the mitochondrial network following localized actin assembly (Moore et al., 2016). Given the prominent role of mitochondrial fragmentation in mitosis, it would be intriguing to determine how actin cycling might act in concert with the established posttranslational modifications and/or interacting partners of DRP1 and its receptors to regulate mitochondrial dynamics in the context of faithful mitotic progression.

## How Mitotic Regulation of Mitochondria Is Linked to Human Diseases?

### Mitochondria-Related Diseases

Defective mitochondrial dynamics either due to fission/fusion imbalance or due to deregulated transport leads to mitochondrial dysfunction and as a consequence to numerous pathological conditions and human disorders, extensively summarized in the review articles (Suárez-Rivero et al., 2016; El-Hattab et al., 2018). However, we still lack sufficient knowledge on if and how defective molecular mechanisms coupling mitochondrial dynamics to mitotic progression can synergistically contribute to several pathological processes, thereby hindering the development of new therapeutic strategies for treatment of these diseases.

Perturbed mitochondrial dynamics and mitochondrial dysfunction have been recognized as leading causes in the development and progression of Parkinson’s disease (Valdinocci et al., 2019), but whether this could also be attributed to the observed defective chromosome segregation has not been explored yet. An siRNA-based high content imaging screening was recently performed to identify genes playing a role in the mechanical properties of mitotic cells and one of the top hits was the Parkinson associated gene *DJ-1/PARK7* (Toyoda et al., 2017), whose Parkinson-associated mutations are known to result in excessively fragmented mitochondria through DRP1 upregulation and increased vulnerability to oxidative stress in neuronal cells (Wang et al., 2012). DJ-1 depletion or loss of its enzymatic activity lead to severe biophysical changes such as reduced force generation and intracellular pressure which are known to be critical for mitotic rounding, a process where the cell changes its architecture in order to create space for spindle assembly and subsequent chromosomal partitioning (Taubenberger et al., 2020). Interestingly, degenerative neurons from Parkinson’s disease patients have paradoxically been described to activate the pRb/E2F1 signaling and undergo mitosis which makes them vulnerable to apoptotic death (Hoglinger et al., 2007), thus suggesting a mechanism on how perturbation of cell division and mitochondrial dynamics regulators could synergistically contribute to the pathogenesis of Parkinson’s disease or similar neurodegenerative diseases. Future studies are needed to expand on this emerging concept.

Another recent study suggests that impairment of mitochondrial dynamics during mitosis could contribute to phenotypes associated with the rare genetic disorder Bloom Syndrome (BS) which is linked to loss of function mutations in the *BLM* gene (Subramanian et al., 2021). Both fibroblasts from BS patients and BML-depleted cells are characterized by extensively fragmented mitochondria in G1 which was associated with failure to degrade Cyclin B1 and subsequently with persistent DRP1 activatory phosphorylation at Ser 616 during late mitosis and re-entry into G1 phase. Given that mitochondrial dynamics perturbation during mitosis has already been correlated to the fine-tuning of Cyclin B1 levels (Pangou et al., 2021), further studies would need to assess the crosstalk between mitochondrial abnormalities and Cyclin B1 dysregulation as a potential therapeutic target pathway in a disease-related context.

### Cancer

Attacking the mitotic machinery of tumor cells through the chemotherapeutic use of mitotic kinase inhibitors and microtubule poisonous agents is a classical anti-cancer strategy, with nonetheless serious side effects and toxicity in patients and is often prone to failure in clinical trials (Tischer and Gergely, 2019). Accumulating evidence suggests that mitochondria represent a promising alternative target in cancer therapy not solely because they are crucial mediators of the intrinsic apoptotic pathway, but also due to the fact that among the central features of carcinogenesis is the adaptation of mitochondrial functions in ways that promote tumor cell proliferation, survival, metastasis and drug resistance (Fulda et al., 2010; Moindjie et al., 2021).

The crossroads of mitotic mitochondrial fission often converge on DRP1-based pathways, suggesting DRP1 as an attractive target for combined anti-cancer strategies (Lima et al., 2018). Large-scale genomic analysis revealed a robust co-expression pattern of DRP1 with cell cycle genes in the majority of the different cancer types tested and subsequent studies that focused on chemosensitive epithelial ovarian cancer models verified that DRP1 expression positively and specifically correlates with genes that promote mitotic transition (Tanwar et al., 2016). The authors suggest that patients displaying the DRP1 signature on their primary tumors, are highly susceptible to relapse after responding to chemotherapy and are predicted to have poor clinical outcome due to aberrant DRP1-driven mitotic progression. Intriguingly, MFF was the only gene among the fission/fusion related genes that was found to strongly follow the genome co-expression profile of DRP1 with mitotic genes, in support of the recent evidence supporting the indispensable role of MFF in the fidelity of chromosome segregation and in providing a survival benefit to highly proliferative cancer cells (Pangou et al., 2021).

Conclusively, identification of new therapeutic targets and intervention strategies based on the crosstalk between the mitotic and the mitochondrial network will potentially prove beneficial in the battle against aneuploidy and aggressive tumor phenotypes.

## Emerging Concepts and Future Perspectives

The mitochondrial network is subjected to dramatic morphological changes during cell cycle transitions. Mitochondria transform from interconnected structures during interphase to highly fragmented ones during mitosis and they need to reestablish an elongated network for the next cell cycle (Figure 1B). The recent technological advances in the field of microscopy have allowed us to dissect in greater detail the dramatic morphological changes that the mitochondria undergo during mitotic progression and to unravel unexpected signaling pathways coupling mitochondrial function to cell division.

The signaling pathways mediating the communication between mitochondria and kinetochore represent a research line that certainly warrants further investigation. Major SAC components such as MPS1, HEC3 and BUB3 have been reported to partially localize at mitochondria but their function there remains unknown (Zhang et al., 2016). Moreover, the transcriptional expression levels of several mitochondrial genes including those involved in the OXPHOS and intrinsic apoptotic pathway have found to be up-regulated upon MPS1 kinase inhibition (Zhang et al., 2016). Likewise, the unexpected localization of exclusively mitochondria-related proteins at the kinetochore has also been described, speaking in favor of the longstanding hypothesis on the existence of a complex mitochondria-to-nucleus retrograde signaling. For example, the DNA helicase TWINKLE which mainly localizes at the mitochondrial nucleoids to regulate mtDNA replication, is enriched at mitotic chromosomes independently of its mitochondrial function and co-localizes with the outer kinetochore protein HEC1/NDC80, a protein indispensable for chromosome congression and SAC activity (Uittenbogaard and Chiaramello, 2016). TWINKLE’s kinetochore function has not yet been dissected but one could speculate that it acts there as part of a novel checkpoint to ensure the temporal coordination of the mtDNA transcription/replication machinery with faithful mitotic progression.

Traditionally, the prevailing dogma is that mitosis is a cellular process with high energy demands and that functional mitochondria are required to provide sufficient ATP amounts to fuel the mitotic machinery (Salazar-Roa and Malumbres, 2017). A recent revolutionary study in single lymphocytic leukemia cells seems to contradict this long-standing theory regarding mitotic bioenergetics and rather argues that ATP synthesis is dramatically decreased during cell division (Kang et al., 2020). The authors demonstrate that mitochondria hyperpolarize at the G2/M transition and they recover during cytokinesis in a manner dependent on the CDK1 activation pattern, as well as that ATP synthesis and ATP levels are reduced in mitosis compared to G2 phase. Moreover, and rather surprisingly in view of our knowledge on the dynamic mitochondrial remodeling throughout cell cycle transitions, the authors claim that mitochondria displayed similar morphologies in interphase and mitosis. Given that mitotic bioenergetics can be differentially regulated depending on the cell type, the synchronization method and the metabolic pathway activated (Kang et al., 2020), further studies are needed to shed light on the mechanistic links between energy homeostasis, mitochondrial dynamics and mitotic progression.

For many years it was thought that cell fate is determined during mitosis, but whether and how this can be coupled to mitochondrial dynamics regulation remained unknown (Wang and Kriegstein, 2020). A recent study attempted to answer these challenging questions using as a model neuron stem cells and monitoring their ability to differentiate into neurons depending on how their mitochondrial network remodels right after mitotic completion (Iwata et al., 2020). Surprisingly, although CDK1-DRP1-mediated mitochondrial fission was equally activated in all cortical progenitors during mitosis and mitochondria remained extensively fragmented for a specific time window frame after mitotic exit, daughter cells destined to self-renew were subjected to mitochondrial fusion, whereas those engaged to differentiate retained high levels of mitochondria fission. It would be very intriguing to dissect in detail whether the molecular links established between mitotic factors and mitochondria-related proteins during mitosis could synergistically act to determine cell fate and reprogramming after mitosis, thus putting the role of mitochondrial and mitotic dysfunction in the development of neurodegenerative diseases into a different perspective.

Since the interactions between mitochondrial dynamics and mitochondrial DNA (mtDNA) integrity and inheritance are tightly interconnected, it is highly possible that deregulation of any of these processes at any stage of the cell cycle can lead to mitochondrial dysfunction and consequently to mitochondria-related diseases. Given the indisputable role of mtDNA mutations in a wide variety of diseases, it is important to consider that there are fundamental differences in the types of mtDNA mutations and in the mtDNA replication models between mitotic and post-mitotic cells. More specifically, in mitotic tissues and cells there is a tendency for both strict and relaxed mtDNA replication alongside with a predisposition for accumulating mtDNA point mutations, whereas in post-mitotic tissues and cells, relaxed mtDNA replication and mtDNA deletions predominate (Lawless et al., 2020). Another important point to consider is that balanced mitochondrial dynamics are a prerequisite for the equal segregation of mtDNA into the daughter cells, and therefore disruption of the mitochondrial morphology during mitotic progression can lead to severe mitochondrial dysfunction and contribute to mitochondrial pathologies (Chapman et al., 2020). For example, disruption of fission results to a hyperfused mitochondrial network that blocks the even distribution of the mitochondrial genome around it, leading to the clustering of nucleoids within the cells (Ban-Ishihara et al., 2013; Ishihara et al., 2015), while on the other hand, disruption of fusion is linked to mtDNA instability and loss of mtDNA copy numbers (Chen et al., 2010; Silva Ramos et al., 2019).

Unbalanced mitochondrial dynamics are often associated with embryonically lethal or severely abnormal developmental phenotypes (Chen et al., 2003, 2015; Davies et al., 2007; Ishihara et al., 2009; The International Mouse Phenotyping Consortium et al., 2016). These phenotypes can be correlated with cumulative damage on mitochondrial functions following several cell divisions, once again underlying the necessity to understand the feedback loops between mitosis and mitochondria as a means to investigate different pathophysiological conditions. Current research focusing on the simultaneous targeting of mitotic and mitochondrial factors has mostly focused on the interconnection between mitotic checkpoints and mitochondrial cell death, but the relationship between the mitotic machinery and mitochondrial dynamics has also started gaining attention (Ruan et al., 2019). Additional studies are undoubtedly essential in order to elucidate the crosstalk between mitochondrial and nuclear genome in an effort to prevent mitochondrial dysfunction which can lead to irreversible damage, chromosomal instability and potentially to human disease.

## Author Contributions

EP and IS: conceptualization, writing, review and editing.

## Conflict of Interest

The authors declare that the research was conducted in the absence of any commercial or financial relationships that could be construed as a potential conflict of interest.

## Publisher’s Note

All claims expressed in this article are solely those of the authors and do not necessarily represent those of their affiliated organizations, or those of the publisher, the editors and the reviewers. Any product that may be evaluated in this article, or claim that may be made by its manufacturer, is not guaranteed or endorsed by the publisher.
